# JCPyV NCCR analysis in PML patients with different risk factors: exploring common rearrangements as essential changes for neuropathogenesis

**DOI:** 10.1186/s12985-020-1295-5

**Published:** 2020-02-11

**Authors:** Maria Rosa Ciardi, Maria Antonella Zingaropoli, Marco Iannetta, Carla Prezioso, Valentina Perri, Patrizia Pasculli, Miriam Lichtner, Gabriella d’Ettorre, Marta Altieri, Antonella Conte, Valeria Pietropaolo, Claudio Maria Mastroianni, Vincenzo Vullo

**Affiliations:** 1 Department of Public Health and Infectious Diseases, Sapienza University of Rome, Piazzale Aldo Moro, 5, 00185 Rome, Italy; 2 Department of System Medicine, Tor Vergata University of Rome, Via Montpellier 1, 00133 Rome, Italy; 3 Infectious Diseases Unit, Sapienza University of Rome, Santa Maria Goretti Hospital, Via Canova, 04100 Latina, Italy; 4 Department of Human Neurosciences, Sapienza University of Rome, Viale dell’Università, 30, 00161 Rome, Italy

**Keywords:** HIV, Multiple sclerosis, CSF, CNS, Disease-modifying therapies

## Abstract

**Background:**

During severe immunosuppression or treatment with specific biological drugs, human polyomavirus JC (JCPyV) may establish a lytic infection in oligodendrocytes, leading to progressive multifocal leukoencephalopathy (PML). Beyond AIDS, which represents the most common predisposing condition, several biological drugs have been associated to the development of PML, such as natalizumab, fingolimod and dimethyl fumarate, which have been showed to increase the risk of PML in the multiple sclerosis (MS) population. JCPyV non-coding control region (NCCR) can be found in two different forms: a virulent neurotropic pathogenic form and a latent non-pathogenic form. The neurotropic forms contain a rearranged NCCR and are typically found in the cerebrospinal fluid, brain or blood of PML patients.

**Case presentation:**

We sequenced and critically examined JCPyV NCCR from isolates detected in the cerebrospinal fluid of four newly diagnosed progressive multifocal leukoencephalopathy patients: two HIV-positive and two HIV-negative multiple sclerosis patients. More complex NCCR rearrangements were observed in the two HIV-positive patients compared to the HIV-negative multiple sclerosis patients with PML.

**Conclusions:**

The comparison of HIV-positive and HIV-negative MS patients with PML, allowed us to evidence the presence of a common pattern of JCPyV NCCR rearrangement, characterized by the deletion of the D-block, which could be one of the initial rearrangements of JCPyV NCCR needed for the development of PML.

## Background

Progressive multifocal leukoencephalopathy (PML) is a severe demyelinating disease of the central nervous system (CNS) caused by the human polyomavirus JC (JCPyV) [1]. Most of the people become seropositive to JCPyV during childhood, and JCPyV can persist and replicate asymptomatically in the urinary tract and in other organs [2]. In immunocompetent subjects, the immune system plays a fundamental role in controlling JCPyV latent infection and the virus is rarely found outside of the urinary tract [3]. During severe immunosuppression or treatment with disease-modifying therapies (DMTs), JCPyV may establish a lytic infection in oligodendrocytes, leading to PML [4, 5]. AIDS represents the most common predisposing condition for PML development [1]. However, the introduction of combination antiretroviral therapies (cART) have reduced the PML risk in HIV-infected patients [1]. To date, several DMTs used in multiple sclerosis (MS) have been associated with PML. Natalizumab, fingolimod and dimethyl fumarate have been associated with an increased risk of developing PML in the MS population [6]. The risk of developing natalizumab-associated PML depends on three factors: the presence of anti-JC antibodies, a long treatment duration (particularly longer than 24 months) and prior immunosuppressive drugs [7]. Although fingolimod and dimethyl fumarate can predispose to PML development, the corresponding risk is significantly lower than natalizumab [8]. Several cases of fingolimod-associated PML have been reported, even excluding carryover patients, coming from a previous natalizumab therapy, and risk factors remain mostly unclear [9, 10]. Age and therapy duration seem to be relevant [8]. Gieselbach et al. have reviewed 19 PML cases associated with fumaric acid esters including dimethyl fumarate in MS [11].

JCPyV genome is characterized by the presence of early and late genes, which encode for non-structural and structural proteins, respectively, and are separated by the non-coding control region (NCCR), which is a key regulatory region of about 400 bp, harboring the origin of viral DNA replication ori, TATA-, TATA-like sequences, several transcription factor binding sites, promoter/enhancer elements and the binding sites for the viral large T-antigen [12]. Among the transcription factor binding sites harbored in the NCCR, the nuclear transcription factor-1 (NF-1) is a cell-specific regulator of JCPyV promoter/enhancer activity [13]; the activating protein 1 (AP-1) is a stimulatory factor involved in JCPyV early gene expression in the absence of viral regulatory proteins [14]; the specificity protein-1 (SP-1) regulates the JCPyV transcription [15]. One of the most remarkable features of the JCPyV is the rearrangement of the promoter/enhancer elements of the NCCR. Indeed, duplications of the promoter/enhancer elements and deletions of the suppressor elements [16] characterize the virulent neurotropic pathogenic form (prototype), which is typically found in the cerebrospinal fluid (CSF), brain and blood of PML patients. Conversely, the non-rearranged form of NCCR is associated to the non-pathogenic form (archetype), which is most frequently detected in urine of healthy subjects as well as PML patients [17].

## Case presentation

The aim of this study was to evaluate and compare the different rearrangements of the JCPyV NCCR sequences detected in CSF samples of four newly diagnosed PML patients with different risk factors: HIV infection and DMTs treatment for MS. Specifically, we describe the cases of two HIV-positive and two HIV-negative MS patients who developed PML. PML diagnosis was performed using magnetic resonance imaging (MRI) and polymerase chain reaction (PCR) that revealed the presence of JCPyV-DNA in CSF samples. For each patient, CSF samples were boiled for 10 min and then centrifuged at 2000 g for 10 min. The resulting supernatants were used directly in molecular biology assays. To detect and quantify the JCPyV genome copy numbers, a 7300 Real-Time quantitative PCR (qPCR) system (Applied Biosystems, USA) was used employing specific primers and probes [18]. Nested PCR with specific primers flanking the NCCR and VP1 regions were performed and PCR products corresponding to JCPyV NCCR and VP1 regions were purified with the QIAquick PCR purification kit, according to QIAGEN protocol [19]. DNA sequencing was performed with a Sanger protocol (Big Dye Terminator Sequencing, Life Technologies), using an ABI 3730 System (Life Technologies, BioFab research s.r.l., Rome, Italy) and the sequences were compared to the archetype CY strain using Sequencing Analysis 5.2 Software (Life Technologies). NCCR sequences have been deposited in Genbank (accession numbers MN241533, MN241534, MN241531, MN241532).

### Case 1

A 31-year-old Caucasian man with MS, after 24 natalizumab infusion was switched to fingolimod due to the persistent high anti-JCPyV antibody index (3.61). After 40 days of fingolimod treatment, the patient developed seizures and visual field defects, leading to hospitalization. The neurological examination revealed: lateral nystagmus to the left, homonymous hemianopia, distal tremor and spasm of the upper limbs. At hospital admission, the patient showed leukopenia (0.83 × 10^9^/L). The brain MRI showed multiple hyperintense areas in T2-weighted and Fluid Attenuated Inversion Recovery (FLAIR) sequences, in the frontal, occipital and parietal lobes of the right hemisphere, with analogue smaller areas in the fronto-parietal lobes of the left hemisphere. Brain lesions showed poor contrast enhancement in T1-weighted sequences.

Suspecting PML, a lumbar puncture (LP) was performed. The CSF composition is reported in Table 1. Furthermore, JCPyV-DNA was detected in CSF (1.62 × 10^6^ IU/mL) by qPCR. Considering clinical presentation, MRI imaging and JCPyV-DNA detection in CSF, the PML diagnosis was confirmed. The patient was treated with mannitol and high dose corticosteroids for 5 days.

**Table 1:** Characteristics of PML patients

	HIV	Risk factor	CSF composition	CD4/CD8 ratio (blood)	CSF JCPyV-DNA IU/mL	NCCR organization
Case 1	negative	natalizumab	glucose: 54 mg/dl proteins: 569 mg/L cell count: 5 cells/μl	0.45	1.62 × 106	A–B*–C–E–F
Case 2	negative	dimethyl fumarate	glucose: 56 mg/dl proteins: 35 mg/L cell count: 2 cells/μl	5.36	4.9 × 105	A–B–C–(D)–E–F
Case 3	positive	HIV infection	glucose: 76 mg/dl proteins: 413 mg/L cell count: 2 cells/μl	0.10	8.97 × 105	A-B-(C)-(E)-(F)-(B)-(C)-(E)-F*
Case 4	positive	HIV infection	glucose: 43 mg/dl proteins: 46 mg/L cell count: 2 cells/μl	0.11	2.34 × 106	A–C*–E*–F*

Letter in brackets indicates the presence of a rearranged block. Asterisk (*) indicates a single nucleotide polymorphism. *CSF* cerebrospinal fluid, *JCPyV* JC polyomavirus, *NCCR* non-coding control region

**Fig. 1:**
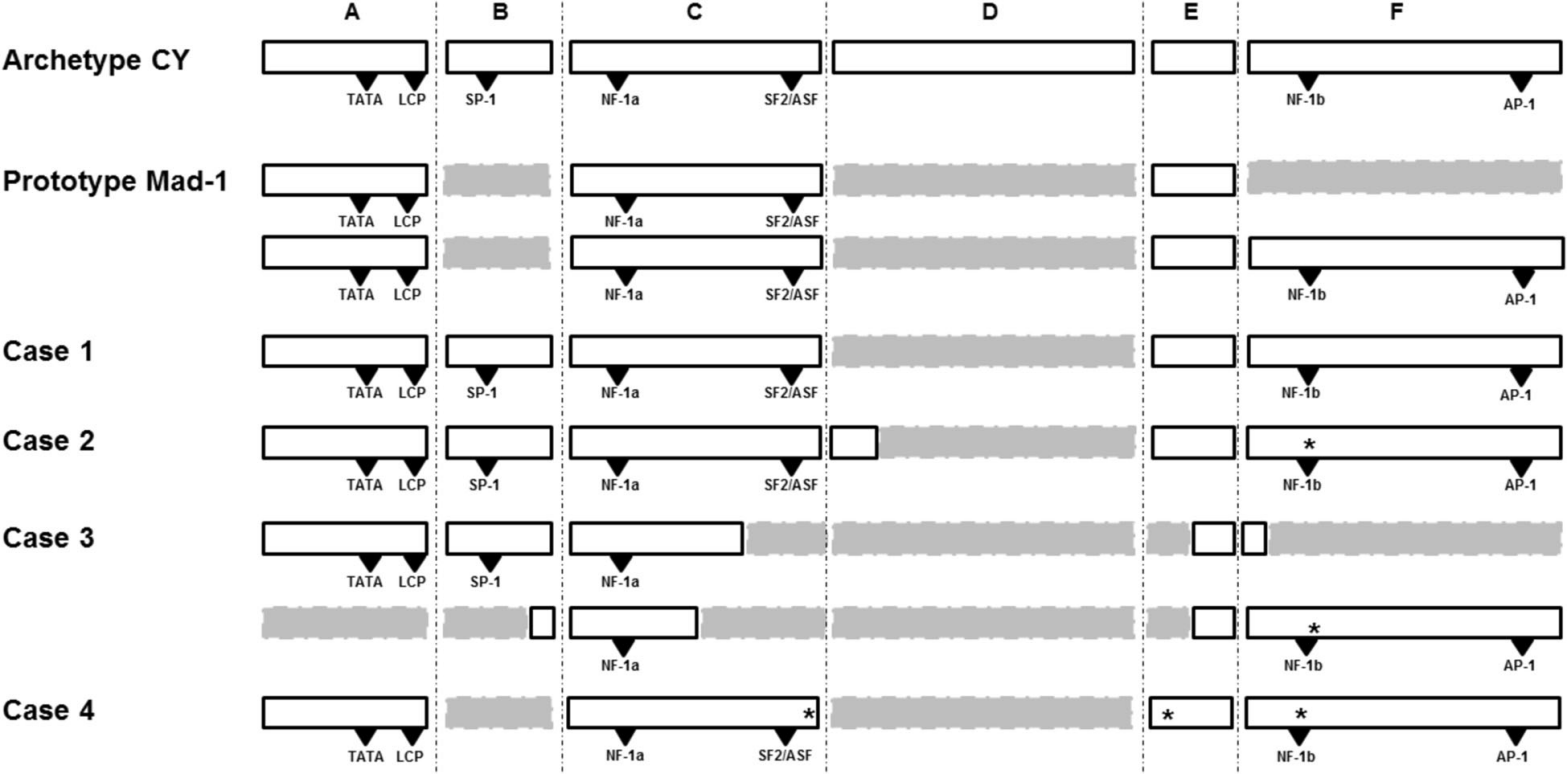
The JCPyV NCCR sequences from CSF of four PML patients. The JCPyVNCCR sits between the viral early gene large T and the late gene agnoprotein and is a 267 base pairs sequence, in the archetype CY strain. The 267 bases are conventionally organized into 6 unequal fragments, namely from A to F: A from 1 to 36, B from 37 to 59, C from 60 to 114, D from 115 to 180, E from 181 to 198, F from 199 to 267 bases [12, 20]. Archetype CY and Mad-1 NCCRs are shown for comparison. Delated blocks are represented in grey with dotted lines. Asterisk (*) represents single nucleotide polymorphism. Transcription factor binding sites are represented by triangles. TATA: tata box; LCP: lytic control element-binding protein; SP-1: specificity protein-1; NF-1a: nuclear transcription factor 1a; SF2/ASF: splicing factor 2/alternative spicing factor; NF-1b: nuclear transcription factor-1b; AP-1: activating protein-1

As reported in Fig. 1, the JCPyV NCCR analysis showed the following block organization: A–B*–C–E–F, with deletion of nucleotides from 117 to 180, corresponding to D-block. Moreover, in B-block, a T to G nucleotide transversion in position 37, corresponding to the Spi-B binding site, was found (which is indicated by the asterisks next to the relative block). VP1 coding region was also sequenced and showed a wild type organization without any mutations.

One month after hospitalization, neurological examination improved significantly, and JCV-DNA became undetectable in CSF sample. Three months after hospitalization, brain MRI showed a reduction in the number of the lesions.

### Case 2

A 41-year-old Caucasian woman with MS started dimethyl fumarate. After 21 months, the patient showed partial disorientation in space and time, nystagmus, left motor syndrome, tremor, dysmetria, paresthesia, speech disorder and cognitive impairment with short term memory loss, which led to dimethyl fumarate discontinuation. At hospital admission, the patient showed leukopenia (0.88 × 10^9^/L). The brain MRI showed hyperintensity in T2 weighted sequences, with homogeneous enhancement in both cortical and subcortical areas of the frontal, parietal and temporal lobes of the left hemisphere. Suspecting PML, LP was performed. The CSF composition is reported in Table 1. JCPyV-DNA was detected in CSF (4.9 × 10^5^ IU/mL) by qPCR. The patient was treated with mannitol and high dose corticosteroids for 5 days.

JCPyV-NCCR was sequenced showing the following organization: A–B–C–(D)–E–F (block in brackets means presence of rearrangements). Specifically, D-block was characterized by a 64-nuclueotide deletion (from 127 to 180), with only 10 remaining nucleotides (from 117 to 126) (Fig. 1). VP1 sequence analysis did not show any mutations.

Three months after hospitalization, JCV-DNA was undetectable in CSF and a brain MRI showed the reduction in the number of the lesions.

### Case 3

A 61-year-old Caucasian man with a medical history including eradicated Hepatitis C virus (HCV) infection, diabetes mellitus-associated neuropathy, leukopenia, previous herpes zoster infection, was admitted to the hospital because of cognitive impairment, motor disfunctions and speech defects. HIV test was performed and was positive. HIV plasma viral load was 2.4 × 10^6^ IU/mL, CD4 cell count was 71 cells/μL, CD8 cell count was 728 cells/μL (CD4/CD8 ratio: 0.10). The neurological examination showed left homonymous hemianopia and astereognosia. A computed tomography (CT) scan of the brain showed hypodensities in the right frontal lobe and in the semioval center. Brain MRI confirmed the presence of T2 weighted and FLAIR sequences hyperintensities in the cortical and subcortical regions of the occipital and parietal right lobes and in the semioval center, without remarkable post-contrast enhancement in T1 weighted sequences. The CSF composition is reported in Table 1. JCPyV-DNA was detected in the CSF (8.97 × 10^5^ IU/mL) by qPCR. Typical clinical and radiological findings and JCPyV-DNA detection in the CSF confirmed the PML diagnosis. The patient received mannitol and steroid therapy. The patient was started with antiretroviral therapy with tenofovir disoproxil fumarate/emtricitabine and dolutegravir.

JCPyV NCCR sequencing showed the following organization: A-B-(C)-(E)-(F)-(B)-(C)-(E)-F*. Specifically, C-block was partially deleted (first 38 nucleotides were maintained, out of 55), D-block was fully deleted (from 117 to 180), E-block (the last 9 nucleotides were maintained, out of 18) and F-block (the first 5 nucleotides were maintained, out of 69) were partially deleted. Furthermore, duplications from nucleotide 54 to 59 of B-block, from 60 to 87 of C-block, from 189 to 198 of E-block and complete of F-block, with a 217 G to A point mutation, were found (Fig. 1). The isolated strain showed one-point mutation (S269F) of VP1 gene, within the VP1 receptor-binding region. One month after hospitalization, a new brain MRI showed disease progression with left hemisphere involvement. The patient was discharged and transferred to a reeducation center.

### Case 4

A 68-year-old Indian man with a six-month history of weight loss, sweating, dry cough, low-grade fever and presence of violet papules on the right leg was admitted to the hospital. Chest x-ray showed thickening in the peribronchial areas associated to diffuse alveolar filling. The patient started antibiotic therapy with intravenous ceftriaxone and oral azithromycin. HIV test was performed and was positive. HIV viral load was 3.8 × 10^5^ IU/mL, CD4 cell count was 35 cells/μL, CD8 cell count was 306 cells/μL (CD4/CD8 ratio: 0.11). The patient started antiretroviral therapy with tenofovir disoproxil fumarate/emtricitabine and raltegravir. CT scan of the brain showed two hypodense subcortical areas in the left temporal lobe without significant mass effect. Brain MRI showed an area of hypointensity in T1-weighted and hyperintensity in T2-weighted sequences of the left occipital and temporal lobes and peritrigonal regions, without remarkable contrast enhancement. The CSF composition is reported in Table 1. JCPyV-DNA was detected in the CSF (2.34 × 10^6^ IU/mL) by qPCR. PML diagnosis was confirmed. Patient was treated with darunavir/ritonavir in addition to the previously indicated antiretroviral treatment.

The JCPyV NCCR sequencing revealed the following block organization: A–C*–E*–F*. Nucleotides 37–59 and 117–180, corresponding to B- and D-block, respectively, were completely deleted. The complete deletions of the B- and D-block implied the loss of both Sp-1 binding sites. Single-nucleotide differences were found in C-block (100 A to C), E-block (183 T to A) and F-block with a characteristic 217 G to A point mutation (Fig. 1). VP1 region sequencing revealed a single mutation causing the amino acid change S267 L involving the receptor-binding region of VP1. One month after hospitalization, a new brain MRI showed disease progression with right hemisphere involvement. The patient was discharged and transferred to a reeducation center.

## Discussion and conclusions

The emergence of PML in MS patients under immunosuppressive therapies and DMTs, such as natalizumab, motivated a search for biological factors contributing to the risk of this serious brain infection [21], such as T-lymphocytes changes [22, 23] and matrix metalloproteinase-9 enzymatic activity [24]. The CNS immune surveillance impairment, the reactivation of latent JCPyV and the blood-brain barrier damage can explain the increased risk of PML development in natalizumab-treated MS patients. Concerning PML in dimethyl fumarate-treated MS patients, only five confirmed cases have been reported, so far [11]. Although the presence of JCPyV in the CNS has been established in all confirmed cases of PML under DMTs, the JCPyV NCCR was not constantly sequenced.

In this study, we characterized JCPyV NCCR sequences detected in the CSF samples of four newly diagnosed PML patients. The JCPyV NCCR analyses revealed a variety of rearranged patterns, which differed from the archetype structure described for the CY strain by Yogo and colleagues [17]. In all cases, the JCPyV NCCR isolates were characterized by partial or complete deletion and duplication of some blocks. Different sequence variations had a similar potential of upregulating viral early gene expression. Similarly to naturally occurring rearranged NCCR of BK virus in kidney transplant patients [25], JCPyV NCCR duplications were found more often in the left blocks A to C, close to the origin of genome replication, while deletions were more frequent in the right end, close to the late genes [26]. A simple hypothesis is that deletions in the proximity of the late genes represent a loss of function, removing a suppressing control transcriptional sequence, whereas duplications in the left end of the NCCR represent a gain of function, increasing activating control sequences that enhance viral replication and gene transcription [27].

As have been previously described by other authors, the TATA box and the first half of the C-block appear to be indispensable for viral replication. This portion of the C-block contains NF-1 binding site that is a cell-specific regulator in JCPyV early transcription and replication [13]. In the C-block, immediately adjacent to the NF-1 site, there is the AP-1 binding site, a stimulatory factor involved in JCPyV early gene expression in the absence of viral regulatory proteins [14]. A consensus recognition sequence for the transcription factor SP-1 exists in the B-block. SP-1 is necessary for simian virus 40 (SV40) transcription [28], and can bind to the SP-1 homology region in BKV [29] as well as the JCV promoter [30].

We observed a more complex NCCR rearrangement in the two HIV-positive patients compared to the two HIV-negative MS patients. Several factor can contribute to the higher grade of JCPyV NCCR rearrangement in HIV-positive patients: the severity of immune impairment, colocalization of HIV and JCPyV in the brain and HIV interaction with JCPyV replication, mediated by viral factors, such as HIV Tat protein [31, 32]. Specifically, HIV Tat is able to increase the viral early gene expression of the JCPyV archetype, to a level that is comparable to the intrinsic activity of the JCPyV strains with a rearranged NCCR [33]. Therefore, it could be speculated an interaction between the two viruses with possible enhancement of JCPyV replication due to the presence of HIV TAT protein, and a more rapid viral evolution with a more complex rearrangement of the NCCR.

In all patients, the JCPyV NCCR was characterized by the complete (case 1, 3 and 4) or partial (case 2) D-block deletion. A higher JCPyV viral load and a lower CD4/CD8 ratio were recorded in those cases characterized by a NCCR with a complete D-block deletion (case 1, 3 and 4). As shown by other authors, this evidence could suggest the role of CD4+ T-cells in controlling viral replication and how the CD8+ T cells cytotoxic response, although important, is not sufficient to prevent PML [34].

Concerning the two HIV-positive patients, we observed JCPyV NCCR rearrangements with both deletions and duplications in case 3 and with deletion only in case 4. As showed by other authors, during NCCR rearrangement, deletions may occur prior to duplications [35]. The analysis of transcription factor binding site modifications within the JCPyV NCCR, revealed that the NF-1 binding site was duplicated in the viral isolate from one HIV-positive patient (case 3), with duplications of other sites such as the AP-1 and SP-1. Interestingly, partial C-block deletion involved the SF2/ASF binding site, a cellular alternative splicing factor, which targets this unique sequence within JCPyV NCCR and strongly inhibits viral transcription and viral propagation in glial cells [36]. The loss of the SF2/ASF binding site could explain the lower viral load found in the case 3, probably due to the impairment of late gene transcription, in which SF2/ASF plays a crucial role for its initiation [37].

Finally, we sequenced the VP1 region of the four isolates. In the two HIV-positive patients point mutations were found in the VP1 coding region (S269F in case 3 and S267 L in case 4). These mutations occurred in VP1 sites for sialic acid binding, changing the virus’s binding properties to its receptor or driving to alternative receptor usage. It has been speculated that mutations within this site may alter the preference of JCPyV capsids from sialylated glycans outside the CNS to non-sialylated glycans inside the CNS, which might explain infection and replication in glial cells [20, 38–41].

JCPyV NCCR rearrangements and VP1 mutations may be independent events that impact distinct elements of JCPyV cellular tropism. However, since VP1 mutations have not been observed in JCPyV with archetype NCCR, VP1 mutations may only arise in JCPyV with the NCCR pathogenic forms [35].

Given the low incidence of PML, the number of patients observed in this preliminary study is very small, thus limiting the general applicability of our findings. Despite this limitation, one of the most relevant evidence of this study is that the complete or partial D-block deletion seems to be an early and crucial rearrangement of NCCR for the development of PML. Interestingly, D-block deletion was the only finding of NCCR sequence rearrangement in the two MS patients with confirmed PML. Differently from HIV late presenters with advanced disease, in MS patients the onset of the immune dysregulation leading to PML, can be easily identified and usually corresponds to the biologic treatment initiation. JCPyV PCR on CSF remains the gold standard for PML diagnosis. Considering the awareness of clinicians about the risk of PML in MS patients under immune modulating therapies, CSF is promptly collected and JCPyV PCR is rapidly performed, when there is such a suspicion. This usually allows the sudden identification of PML in the early stages of the disease. Consequently, the detection of JCPyV-DNA in the CSF early in the history of PML can reveal the presence of a NCCR at the beginning of the sequence of rearrangement events.

According to these considerations, we speculate that D-block deletion observed as the only rearrangement in the two MS patients, but also present in the two HIV late presenters, could represent one of the early but crucial steps in the complex series of NCCR rearrangements leading to PML. Considering the limited number of cases reported here, it is difficult to establish a correlation between the outcome and the type of rearrangements observed in the JCPyV NCCR. Moreover, MS patients showed a better prognosis, probably because of the discontinuation of the DMT and consequent immune reconstitution. Indeed, after DMT removal, MS patients with PML can experience an immune reconstitution syndrome (IRIS), which is similar to PML-IRIS occurring in some HIV patients after cART initiation [1].

One limitation of our study is represented by the use of the classical Sanger method for sequencing JCPyV NCCR. Several innovative techniques for identifying and sequencing viral strains are now available. As showed by Seppälä et al., next generation sequencing (NGS) has the ability to characterize JCPyV NCCR with more accuracy, identifying minority variants and multiple strains with different rearrangements within the same individual [42]. Although in this study we used the Sanger method, NGS represent a promising approach for identifying JCPyV minority variants and understanding viral evolution.

These preliminary data could stimulate additional researches on wider cohorts involving HIV-positive and HIV-negative patients to determine whether the changes observed in our patients are consistently present. Moreover, tissue distribution of JCPyV in healthy subjects and patients with HIV or MS needs to be assessed, as well as longitudinal studies in patients under DMTs with PML risk, in order to clarify the pathogenesis of JCPyV reactivation and PML onset. These translational approaches should be associated with improved methods for detection of low JCPyV viral load in the CSF and blood samples, which may help in better defining the risk of developing PML in susceptible categories of patients.

## Data Availability

All the data and materials used in this report are included in the manuscript.

## Abbreviations

AP-1: Activating protein 1
CSF: Cerebrospinal fluid
CT: Computed tomography
DMTs: Disease-modifying therapies
FLAIR: Fluid Attenuated Inversion Recovery
JCPyV: JC polyomavirus
LP: Lumbar puncture
MRI: Magnetic resonance imaging
MS: Multiple sclerosis
NCCR: Non-coding control region
NF-1: Nuclear transcription factor-1
PML: Progressive multifocal leukoencephalopathy
SP-1: Specificity protein-1

## References

[1] Iannetta Marco, Ciardi Maria Rosa, Zingaropoli Maria Antonella, D'Abramo Alessandra, Oliva Alessandra, Mastroianni Claudio Maria, Vullo Vincenzo (2016). HIV-associated progressive multifocal leukoencephalopathy: current perspectives. Neurobehavioral HIV Medicine.

[2] Grinnell BW, Padgett BL, Walker DL (1983). Distribution of nonintegrated DNA from JC papovavirus in organs of patients with progressive multifocal leukoencephalopathy. J Infect Dis.

[3] Koralnik IJ, Boden D, Mai VX, Lord CI, Letvin NL (1999). JC virus DNA load in patients with and without progressive multifocal leukoencephalopathy. Neurology.

[4] Berger JR, Concha M (1995). Progressive multifocal leukoencephalopathy: the evolution of a disease once considered rare. J Neuro-Oncol.

[5] Koralnik IJ (2004). New insights into progressive multifocal leukoencephalopathy. Curr Opin Neurol.

[6] Mills EA, Mao-Draayer Y (2018). Understanding progressive multifocal Leukoencephalopathy risk in multiple sclerosis patients treated with Immunomodulatory therapies: a Bird’s eye view. Front Immunol.

[7] Bloomgren G, Richman S, Hotermans C, Subramanyam M, Goelz S, Natarajan A (2012). Risk of natalizumab-associated progressive multifocal leukoencephalopathy. N Engl J Med.

[8] Berger Joseph R., Cree Bruce A., Greenberg Benjamin, Hemmer Bernhard, Ward Brian J., Dong Victor M., Merschhemke Martin (2018). Progressive multifocal leukoencephalopathy after fingolimod treatment. Neurology.

[9] Gyang Tirisham V., Hamel Johanna, Goodman Andrew D., Gross Robert A., Samkoff Lawrence (2016). Fingolimod-associated PML in a patient with prior immunosuppression. Neurology.

[10] Nishiyama S, Misu T, Shishido-Hara Y, Nakamichi K, Saijo M, Takai Y (2018). Fingolimod-associated PML with mild IRIS in MS: a clinicopathologic study. Neurol Neuroimmunol Neuroinflammation.

[11] Gieselbach R-J, Muller-Hansma AH, Wijburg MT, de Bruin-Weller MS, van Oosten BW, Nieuwkamp DJ (2017). Progressive multifocal leukoencephalopathy in patients treated with fumaric acid esters: a review of 19 cases. J Neurol.

[12] McIlroy D, Halary F, Bressollette-Bodin C (2019). Intra-patient viral evolution in polyomavirus-related diseases. Philos Trans R Soc Lond Ser B Biol Sci.

[13] Chen NN, Khalili K (1995). Transcriptional regulation of human JC polyomavirus promoters by cellular proteins YB-1 and Pur alpha in glial cells. J Virol.

[14] Sadowska B, Barrucco R, Khalili K, Safak M (2003). Regulation of human polyomavirus JC virus gene transcription by AP-1 in glial cells. J Virol.

[15] Romagnoli L, Sariyer IK, Tung J, Feliciano M, Sawaya BE, Del Valle L (2008). Early growth response-1 protein is induced by JC virus infection and binds and regulates the JC virus promoter. Virology.

[16] Fedele CG, Ciardi MR, Delia S, Contreras G, Perez JL, De Oña M (2003). Identical rearranged forms of JC polyomavirus transcriptional control region in plasma and cerebrospinal fluid of acquired immunodeficiency syndrome patients with progressive multifocal leukoencephalopathy. J Neuro-Oncol.

[17] Yogo Y, Kitamura T, Sugimoto C, Ueki T, Aso Y, Hara K (1990). Isolation of a possible archetypal JC virus DNA sequence from nonimmunocompromised individuals. J Virol.

[18] Bellizzi A, Anzivino E, Rodio DM, Cioccolo S, Scrivo R, Morreale M (2013). Human Polyomavirus JC monitoring and noncoding control region analysis in dynamic cohorts of individuals affected by immune-mediated diseases under treatment with biologics: an observational study. Virol J.

[19] Pietropaolo V, Videtta M, Fioriti D, Mischitelli M, Arancio A, Orsi N (2003). Rearrangement patterns of JC virus noncoding control region from different biological samples. J Neuro-Oncol.

[20] Dubensky TW, Freund R, Dawe CJ, Benjamin TL (1991). Polyomavirus replication in mice: influences of VP1 type and route of inoculation. J Virol.

[21] Pavlovic D, Patera AC, Nyberg F, Gerber M, Liu M (2015). Progressive multifocal Leukeoncephalopathy consortium. Progressive multifocal leukoencephalopathy: current treatment options and future perspectives. Ther Adv Neurol Disord.

[22] Iannetta Marco, Zingaropoli Maria Antonella, Bellizzi Anna, Morreale Manuela, Pontecorvo Simona, D’Abramo Alessandra, Oliva Alessandra, Anzivino Elena, Lo Menzo Sara, D’Agostino Claudia, Mastroianni Claudio Maria, Millefiorini Enrico, Pietropaolo Valeria, Francia Ada, Vullo Vincenzo, Ciardi Maria Rosa (2016). Natalizumab Affects T-Cell Phenotype in Multiple Sclerosis: Implications for JCV Reactivation. PLOS ONE.

[23] Zingaropoli Maria A., Iannetta Marco, Pontecorvo Simona, Anzivino Elena, Prezioso Carla, Rodio Donatella Maria, Morreale Manuela, D’Abramo Alessandra, Oliva Alessandra, Lichtner Miriam, Cortese Antonio, Frontoni Marco, Pietropaolo Valeria, Francia Ada, Mastroianni Claudio M., Vullo Vincenzo, Ciardi Maria R. (2018). JC Virus-DNA Detection Is Associated with CD8 Effector Accumulation in Peripheral Blood of Patients with Multiple Sclerosis under Natalizumab Treatment, Independently from JC Virus Serostatus. BioMed Research International.

[24] Iannetta M, Zingaropoli MA, Latronico T, Pati I, Pontecorvo S, Prezioso C, et al. Dynamic changes of MMP-9 plasma levels correlate with JCV reactivation and immune activation in natalizumab-treated multiple sclerosis patients. Sci Rep [Internet]. 2019 Jan 22 [cited 2019 Jul 28];9. Available from: https://www.ncbi.nlm.nih.gov/pmc/articles/PMC6342994/10.1038/s41598-018-36535-5 PMC6342994 30670793

[25] Gosert R, Rinaldo CH, Funk GA, Egli A, Ramos E, Drachenberg CB (2008). Polyomavirus BK with rearranged noncoding control region emerge in vivo in renal transplant patients and increase viral replication and cytopathology. J Exp Med.

[26] Vacante DA, Traub R, Major EO (1989). Extension of JC virus host range to monkey cells by insertion of a simian virus 40 enhancer into the JC virus regulatory region. Virology.

[27] Daniel AM, Swenson JJ, Mayreddy RP, Khalili K, Frisque RJ (1996). Sequences within the early and late promoters of archetype JC virus restrict viral DNA replication and infectivity. Virology.

[28] Dynan WS, Chervitz SA (1989). Characterization of a minimal simian virus 40 late promoter: enhancer elements in the 72-base-pair repeat not required. J Virol.

[29] Markowitz RB, Tolbert S, Dynan WS (1990). Promoter evolution in BK virus: functional elements are created at sequence junctions. J Virol.

[30] Chowdhury M, Taylor JP, Chang CF, Rappaport J, Khalili K (1992). Evidence that a sequence similar to TAR is important for induction of the JC virus late promoter by human immunodeficiency virus type 1 tat. J Virol.

[31] Stettner MR, Nance JA, Wright CA, Kinoshita Y, Kim W-K, Morgello S (2009). SMAD proteins of oligodendroglial cells regulate transcription of JC virus early and late genes coordinately with the tat protein of human immunodeficiency virus type 1. J Gen Virol.

[32] Tada H, Rappaport J, Lashgari M, Amini S, Wong-Staal F, Khalili K (1990). Trans-activation of the JC virus late promoter by the tat protein of type 1 human immunodeficiency virus in glial cells. Proc Natl Acad Sci U S A.

[33] Gosert R, Kardas P, Major EO, Hirsch HH (2010). Rearranged JC virus noncoding control regions found in progressive multifocal leukoencephalopathy patient samples increase virus early gene expression and replication rate. J Virol.

[34] Gheuens S, Bord E, Kesari S, Simpson DM, Gandhi RT, Clifford DB (2011). Role of CD4+ and CD8+ T-cell responses against JC virus in the outcome of patients with progressive multifocal leukoencephalopathy (PML) and PML with immune reconstitution inflammatory syndrome. J Virol.

[35] Reid CE, Li H, Sur G, Carmillo P, Bushnell S, Tizard R (2011). Sequencing and analysis of JC virus DNA from natalizumab-treated PML patients. J Infect Dis.

[36] Sariyer IK, Khalili K (2011). Regulation of human neurotropic JC virus replication by alternative splicing factor SF2/ASF in glial cells. PLoS One.

[37] Uleri E, Regan P, Dolei A, Sariyer IK (2013). SF2/ASF binding region within JC virus NCCR limits early gene transcription in glial cells. Virol J.

[38] Sunyaev SR, Lugovskoy A, Simon K, Gorelik L (2009). Adaptive mutations in the JC virus protein capsid are associated with progressive multifocal leukoencephalopathy (PML). PLoS Genet.

[39] Bauer PH, Bronson RT, Fung SC, Freund R, Stehle T, Harrison SC (1995). Genetic and structural analysis of a virulence determinant in polyomavirus VP1. J Virol.

[40] Bauer PH, Cui C, Liu WR, Stehle T, Harrison SC, DeCaprio JA (1999). Discrimination between sialic acid-containing receptors and pseudoreceptors regulates polyomavirus spread in the mouse. J Virol.

[41] Freund R, Garcea RL, Sahli R, Benjamin TL (1991). A single-amino-acid substitution in polyomavirus VP1 correlates with plaque size and hemagglutination behavior. J Virol.

[42] Seppälä Hanna M, Helanterä Ilkka T, Laine Pia K S, Lautenschlager Irmeli T, Paulín Lars G, Jahnukainen Timo J, Auvinen Petri O V, Auvinen Eeva (2017). Archetype JC Polyomavirus (JCPyV) Prevails in a Rare Case of JCPyV Nephropathy and in Stable Renal Transplant Recipients With JCPyV Viruria. The Journal of Infectious Diseases.

